# Commentary on Wittes et al: Aspirin for primary prevention of CV events – Rationally robust? Statistically significant? Clinically convincing?

**DOI:** 10.1177/17407745251324865

**Published:** 2025-04-01

**Authors:** John GF Cleland, Danyaal Anzar

**Affiliations:** 1 British Heart Foundation Cardiovascular Research Centre, School of Cardiovascular and Metabolic Health, University of Glasgow, Glasgow, UK; 2 University of Glasgow, Glasgow, UK

Wittes et al.^1^ provide a thoughtful perspective on how to interpret the totality of evidence from randomised trials but it is not clear that they follow their own advice. They write, very sensibly, that

Inference about treatment should not be based on undue reliance on individual small trials, meta-analyses of small trials, subgroups, or post-hoc analyses. Failure to follow these principles can lead to conclusions inconsistent with the totality of evidence and to inappropriate recommendations made by guideline committees.

The authors then go on to single out the Aspirin in Reducing Events in the Elderly (ASPREE) trial,^2,3^ for criticism, suggesting it is inconsistent with other trials. This criticism may reflect the authors’ opinions, but the totality of evidence suggests otherwise. Most randomised trials of aspirin for primary prevention, including ASPREE, were stopped for futility or fatigue. None of the 10 trials included in the meta-analyses of aspirin quoted by the authors showed clear benefit^1^ (see Table 1); only one had a marginally positive result. Many trials were conducted last century and in highly selected populations (for instance, male physicians). Background risk factors and therapies have changed. At least one large trial showed an increased risk of myocardial infarction with aspirin but has been excluded from meta-analyses for no clear reason.^4^ Surely, ASPREE is one of the most relevant trials of aspirin for contemporary clinical practice.^5^

**Table 1.** Summary results of large, randomised trials comparing aspirin with placebo for the primary prevention of cardiovascular events.

Trial and years initiated and published	N =	Patient-years (1000)	Aspirin dose (mg/day)	Primary endpoint	Completed as planned	Reason for stopping	Significant for pre-specified primary endpoint?	Deaths prevented per 1000 patient-years	CV Deaths prevented per 1000 patient-years	Disabling stroke caused or prevented per 1000 patient-years^a^	Disability or death prevented per 1000 patient-years	People who did not benefit per decade of aspirin therapy
British Doctors 1978-1988	5139	∼28	500	CV Events	No	Fatigue?	No	↓2 (ns)	↓< 1 (ns)	↑**1 (p** **<** **0.05)**	< 1 (ns)	∼99.9%
US Physicians 1982-1988	22,071	∼110	325 alt.	CV Mortality	No	Futility^5^	No	↓< 1 (ns)	0	↑< 1 (ns)	0	∼100%
Thrombosis Prevention Trial 1984-1998	2540	∼16	75	IHD Events	No	Fatigue?	No	0	0	0	0	∼100%
HOT 1992-1998	18,790	∼71	75	CV Events	No	Fatigue?	No	↓< 1(ns)	↓< 1 (ns)	0	0	∼100%
Primary Prev. Proj. 1994-2001	4495	∼16	100	CV Events	No	Prior Trials	No	↓2 (ns)	↓2 (ns)	0	0	∼99.9%
Women’s Health 1992-2005	39,876	∼395	100 alt.	CV Events	Yes?	NA	No	↓< 1 (ns)	↓< 1 (ns)	0^b^	0	∼99.9%
ASCEND 2005-2018	15,480	∼114	100	CV Events	Yes?	NA	**Yes (p** **=** **0.01)**	↓1 (ns)	↓< 1 (ns)	0	0	∼99.9%
ARRIVE 2007-2018	12,546	∼63	100	CV Events	No	Fatigue?	No	0	0	0	0	∼100%
ASPREE 2010-2018	19,114	∼88	100	Disability-Free Survival	No	Futility	No	↑**2 (p** **<** **0.01)**	↑< 1 (ns)	0	0	∼100%
International Polycap Study 2012-2020	5713	∼25	75	CV Events	Yes?	NA	No	↓2 (ns)	↓1 (ns)	0^c^	< 2 (ns)	∼99.8%

∼approximately, often calculated from patient numbers and duration of follow-up. Alt. = alternate days. NA = not applicable. Yes? = probably after one or more amendments. Fatigue? = trial appeared to run out of time and/or resources usually because event rates were much lower than expected.

a In the absence of a specific report on disabling strokes, if overall stroke rates were similar on aspirin and placebo, it was assumed there was no difference in disabling stroke.

b Overall ∼0.2 fewer strokes per 1000 patient-years (p = 0.04) – unlikely that disabling strokes were reduced significantly.

c Overall ∼1.3 fewer strokes per 1000 patient-years (p < 0.05) – unlikely that disabling strokes were reduced significantly. See reference 1 for references to trials in this table. Reference 15 provides a full explanation for why the US Physicians’ Trial was stopped.

The authors suggest that guidelines have been unduly influenced by ASPREE, because it ‘…. did not provide evidence that aspirin showed no benefit…’… I am sure the authors did not mean to suggest that all treatments should be considered effective until there is evidence to the contrary. Only treatments with conclusive evidence of a worthwhile clinical benefit should be recommended with confidence to patients. There is no convincing evidence that long-term treatment with aspirin is effective for primary or secondary prevention of cardiovascular events, dementia, cancer or any other disease at any level of cardiovascular risk (see Table 1).^2–4,6^ Indeed, long-term aspirin therapy may be deleterious.^2–4,6,7^

The whole hypothesis that anti-thrombotic therapy reduces the risk of vascular events is founded on the belief that myocardial infarction and stroke are driven mainly by thrombotic arterial occlusion. Cardiologists may have succumbed to a hypothesis that is not well substantiated. Intraplaque haemorrhage leading to rupture may be a common cause of vascular occlusion, with thrombosis only a secondary phenomenon.^7,8^ Any benefit from aspirin in reducing thrombosis may be negated by an increase in plaque haemorrhage. Only in the few weeks following plaque rupture and endothelial loss may the benefits of reducing thrombosis outweigh the harms of increasing plaque haemorrhage.^4,7,8^

Although there is some evidence that aspirin does reduce clinically overt cardiovascular events, there is no evidence that this translates into a reduction in disability or death. One explanation for this anomaly is that aspirin might change the presentation of vascular occlusions rather reducing them. Many vascular events are not diagnosed (‘silent’) or present as sudden death; aspirin might increase the risk of both.^7,8^

As for an effect on reducing the risk of cancer, this is also not based on strong evidence. For instance, Baron et al.^9^ found that for patients with a recently treated colonic adenoma, fewer new adenomas developed in those randomised to aspirin 81 mg/day but not to 325 mg/day, but more patients randomised to aspirin developed cancer (16 on low-dose and 12 on high-dose) compared to placebo (n = 7). Those randomised to aspirin also tended to have more strokes (p = 0.06). The CAPPS-2 trial,^10,11^ comparing aspirin 600 mg/day to placebo in patients with a proven or suspected genetic mutation predisposing to some cancers, followed patients for up to 20 years. However, of 861 patients randomised 522 (61%) were lost to follow-up by 10 years, at which time aspirin had no significant effect on the rate of colon or other cancers. Only in the year after this massive loss to follow-up were differences in cancer rates observed.^10^ These finding are hard to interpret. The ASCEND trial reported 1784 incident cancers (including 484 gastro-intestinal) with no difference between placebo and aspirin.^12^ There were 2865 incident cancer cases in the Women’s Health Study, the largest randomised trial of primary prevention aspirin. Aspirin neither reduced cancer incidence nor mortality.^13^ In the ASPREE trial, aspirin also had no effect on the overall risk of cancer but increased the number presenting with metastatic disease and cancer deaths.^14^ More recently, a trial of aspirin in patients (mostly women) treated for breast cancer was stopped prematurely due to a strong trend to harm among those assigned to aspirin.^15^ As with cardiovascular disease, it is important to consider all of the data.

When interpreting the clinical importance of a treatment effect in trials, it is the absolute rather than relative difference that is important; confidence intervals and p-values are not measures of the size of benefit, just the probability that suspected differences did not occur by chance. Relative differences, often expressed as a hazard ratio, may look impressive when the absolute event rate is low. A hazard ratio of 0.87 (the effect found in the quoted updated meta-analysis) on a background event rate of 1% per year (10% per decade) would mean giving aspirin to about 800 people for a year to prevent one event, with no certainty that this will reduce disability or mortality and with the risk of serious harm.

If there is any benefit at all from aspirin for primary prevention of cardiovascular events or cancer, it is small. Rather than persevering with a failed hypothesis and inflicting treatments of dubious value on patients, is it not time to abandon use of long-term antiplatelet therapy altogether? Long-term aspirin therapy carries a risk of serious harms, including intracerebral haemorrhage, disabling stroke and accelerating cancer progression. Perseveration (doing the same thing over and over again) but expecting different results is a definition of insanity widely (mis-)attributed to Einstein. Perhaps research resources should now focus on the benefits of stopping aspirin for secondary prevention after the acute event has settled?
